# Lipoteichoic acid biosynthesis by *Staphylococcus aureus* is controlled by the MspA protein

**DOI:** 10.1128/mbio.01512-24

**Published:** 2024-07-22

**Authors:** Dora Bonini, Seána Duggan, Alaa Alnahari, Tarcisio Brignoli, Henrik Strahl, Ruth C. Massey

**Affiliations:** 1 School of Cellular and Molecular Medicine, University of Bristol, Bristol, United Kingdom; 2 MRC Centre for Medical Mycology, University of Exeter, Exeter, United Kingdom; 3 Department of Biological Sciences, University of Jeddah, Jeddah, Saudi Arabia; 4 Department of Biosciences, Università degli Studi di Milano, Milan, Italy; 5 Centre for Bacterial Cell Biology, Biosciences Institute, Newcastle University, Newcastle upon Tyne, United Kingdom; 6 School of Microbiology, University College Cork, Cork, Ireland; 7 APC Microbiome Ireland, University College Cork, Cork, Ireland; Universite de Geneve, Geneva, Switzerland

**Keywords:** *Staphylococcus aureus*, virulence, LTA, MspA

## Abstract

*Staphylococcus aureus* produces a plethora of virulence factors critical to its ability to establish an infection and cause disease. We have previously characterized a small membrane protein, MspA, which has pleiotropic effects on virulence and contributes to *S. aureus* pathogenicity *in vivo*. Here we report that *mspA* inactivation triggers overaccumulation of the essential cell wall component, lipoteichoic acid (LTA), which, in turn, decreases autolytic activity and leads to increased cell size due to a delay in cell separation. We show that MspA directly interacts with the enzymes involved in LTA biosynthesis (LtaA, LtaS, UgtP, and SpsB), interfering with their normal activities. MspA, in particular, interacts with the type I signal peptidase SpsB, limiting its cleavage of LtaS into its active form. These findings suggest that MspA contributes to maintaining a physiological level of LTA in the cell wall by interacting with and inhibiting the activity of SpsB, thereby uncovering a critical role for the MspA protein in regulating cell envelope biosynthesis and pathogenicity.

**IMPORTANCE:**

The *S. aureus* cell envelope, comprising the cytoplasmic membrane, a thick peptidoglycan layer, and the anionic polymers lipoteichoic acid and wall teichoic acids, is fundamental for bacterial growth and division, as well as being the main interface between the pathogen and the host. It has become increasingly apparent that the synthesis and turnover of cell envelope components also affect the virulence of *S. aureus*. In this study, we show that MspA, an effector of *S. aureus* virulence, contributes to the maintenance of normal levels of lipoteichoic acid in the cell wall, with implications on cell cycle and size. These findings further our understanding of the connections between envelope synthesis and pathogenicity and suggest that MspA represents a promising target for the development of future therapeutic strategies.

## INTRODUCTION

*Staphylococcus aureus* is a major human opportunistic pathogen (1) associated with more than 1 million deaths globally in 2019 (2). It asymptomatically colonizes 20%–30% of the population, which represents a risk factor for later development of disease (3, 4). *S. aureus* causes a range of infections from mild skin and soft tissue presentations such as impetigo to invasive diseases including pneumonia, endocarditis, osteomyelitis, and bacteremia (5). Key to its ability to cause this range of infections is the production of virulence factors, including adhesins, cytolytic toxins, and immune evasins (6, 7). The regulation and utilization of such factors are complex and dependent upon the ability of the bacterium to sense and respond to their environment. Critical to all of this is the bacterial cell envelope, which represents the part of the bacteria directly in contact with the host. While maintaining structural integrity and enabling growth and division, the cell envelope is also a main defense barrier to components of the innate immune system such as cationic antimicrobial peptides (AMPs) and fatty acids (8), as well as the target site of clinically relevant antibiotics oxacillin, vancomycin, and daptomycin (9). As such, the regulation of synthesis and renewal of the envelope components are of great interest both for understanding *S. aureus* pathogenicity and for the development of new therapeutics.

The *S. aureus* envelope comprises the phospholipid bilayer surrounded by the highly cross-linked peptidoglycan sacculus (10). The cell wall sacculus is interwoven with two types of anionic glycopolymers, wall teichoic acids (WTA), chains of ribitol-phosphate covalently linked to peptidoglycan, and lipoteichoic acids (LTAs), chains of glycerol-phosphate anchored to the membrane via a diglucosyl‐diacylglycerol (Glc_2_DAG) lipid anchor (11, 12). WTA and LTA have overlapping roles in regulating cell division, autolysin activity, cation homeostasis, and affinity to AMPs (13). Some of these properties are further regulated by addition of D-alanine residues to the repeating units of both WTA and LTA by proteins encoded by the *dlt* operon, which reduces the overall negative charge of the polymers (14).

Although they share some characteristics, these teichoic acids also perform unique and separate functions (15). While *S. aureus* is viable in laboratory conditions without WTA (16), deletion of the LTA synthase *ltaS* is lethal for growth at 37°C, as cells cannot withstand osmotic pressure (17) and show aberrant septum formation and division (18). Mutants of *ltaS* have also been documented to acquire suppressor mutations which bypass the requirement for LTA via different mechanisms, such as reducing internal turgor pressure (19–21). The LTA pathway includes GtaB and PgcA, which synthesize UDP-glucose; UgtP (also named YpfP), which transfers glucose to diacylglycerol; LtaA, which facilitates the flipping of the Glc_2_DAG anchor from the cytoplasmic to the outer leaflet of the membrane (22); and LtaS, which catalyzes the polymerization of glycerol phosphate on the anchor (18). Other cell envelope- associated enzymes with more generalized activities, such as the type I signal peptidase SpsB, are also involved in the LTA synthesis pathway, where it cleaves LtaS, releasing the synthase’s extracellular catalytic domain from the five transmembrane helices (23, 24). Additionally, MprF, which catalyses lysinylation of anionic membrane lipids, has been shown to stimulate LTA synthesis via LtaS in both *Bacillus subtilis* and *S. aureus* (25).

We previously identified and characterized a small membrane protein called MspA (26, 27). Inactivation of *mspA* in both the MRSA strain JE2 and the MSSA strain SH1000 was shown to cause a significant decrease in phenol soluble modulins toxin production and cytotoxicity due to downregulation of the accessory gene regulatory system. The *mspA* mutant was shown to have a reduced content of the membrane carotenoid pigment staphyloxanthin, to be more susceptible to components of the innate immune response such as fatty acids and AMPs, and to have decreased membrane stability when challenged with the detergent SDS. Interestingly, both systems for uptake (IsdC, IsdB, and FhuC) and export (HrtAB) of heme, the major iron source for *S. aureus* during infection, were more abundant in the *mspA* mutant compared to the wild type. The mutant also had increased levels of intracellular iron, suggesting dysregulation of iron homeostasis. These pleiotropic effects observed *in vitro* resulted in attenuation of the mutant in superficial and systemic infection mouse models (27).

Given the predicted location of MspA and the pleiotropic effects its inactivation has on virulence and pathogenicity, we hypothesized that MspA could have a structural role, contributing to the synthesis of the membrane or cell wall or supporting the stability of proteins involved in such processes. Therefore, virulence defects showed by the mutant could be a downstream consequence of perturbations in cellular structural integrity. Here we show that inactivation of *mspA* leads to an increase in cell size and a delay in cell cycle progression due to overaccumulation of LTA. We demonstrate that MspA interacts with the LTA-associated enzymes SpsB, UgtP, LtaA, and LtaS and suggest that MspA maintains physiological LTA levels in the cell wall by interfering with SpsB’s ability to process LtaS. With such a critical role in cell envelope biosynthesis and subsequently in pathogenesis, we propose that the MspA protein represents a major target for future therapeutic intervention.

## RESULTS

### The MspA protein affects cell size and cell cycle progression

**Fig 1:**
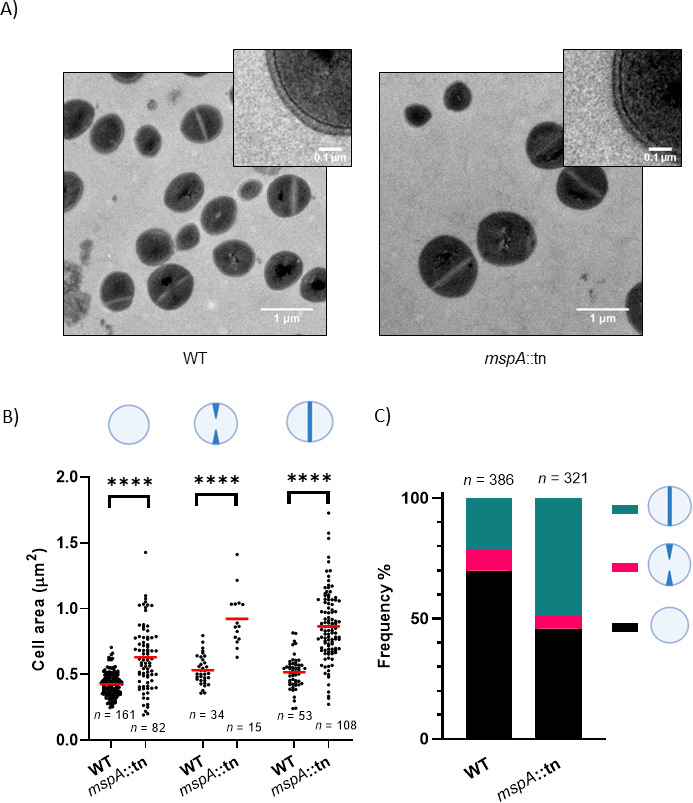
MspA-deficient cells are larger and cell separation is delayed. (**A**) Transmission electron microscopy micrographs of SH1000 wild-type (WT) and isogenic *mspA* transposon mutant (*mspA*::tn) cells. (**B**) Measurements of cell area of wild-type and *mspA*::tn cells in each cell division stage. (From left to right) Non-dividing cells without any visible septum (Welch’s *t*-test), dividing cells with an incomplete septum (Welch’s *t*-test), and dividing cells with a fully formed complete septum (Welch’s *t*-test). Red lines indicate the mean. (**C**) Proportion of cells in the three phases of cell division. The total number of analyzed cells is indicated on top of the stack graph. ****P < 0.0001.

The *S. aureus* small membrane protein MspA has been shown to have a large impact on virulence, pathogenicity, and membrane stability (27). As it is largely embedded in the membrane and lacks any annotated functional domains, we hypothesized that it could have a structural role and could support envelope synthesis directly or stabilize proteins involved in membrane and cell wall biogenesis. To test this, we first investigated if mutation of *mspA* caused any visible alterations to the membrane or cell wall by performing transmission electron microscopy (TEM). Our first observation was that many of the MspA-deficient cells were noticeably larger than the wild-type cells in both the SH1000 (Fig. 1A) and JE2 backgrounds (Fig. S1). We decided to focus further work on the SH1000 background, where we have confirmed the increased size phenotype by quantifying the cell area, which we used as an approximate measure for cell size (Fig. 1B). While there were no major differences in the envelope architecture between most of the wild-type and mutant cells (Fig. 1A), a small number of MspA-deficient cells (5 out of >300) had irregular septa which formed before daughter cell separation and were not oriented perpendicular to the previous division plane (Fig. S2). We did not observe these abnormalities in the wild-type population, having examined an equivalent number of cells. To assess whether the increase in cell size affected the mutant throughout the cell cycle or if it was specific to a stage, we classified cells in three phases: (i) non-dividing cells without any visible septum, (ii) actively dividing cells with incomplete septa, or (iii) divided cells with complete fully formed septa. The size of the MspA-deficient cells was significantly increased in all the cell cycle stages compared to the wild type (Fig. 1B). We also noticed that the proportion of cells in each of the cell cycle stages varied between the wild type and the mutant (Fig. 1C). In particular, the proportion of cells with a fully formed septum was more than double for the MspA-deficient cells (48.9% vs 21.5% of the total number of cells analyzed). The fraction of cells in each division stage is proportional to the time that cells spend in that stage (28). Therefore, a higher proportion of cells with a fully formed septum suggests that lack of MspA triggers a delay in daughter cell separation after septum formation. It has been shown that *S. aureus* cells with a complete septum elongate before splitting into two daughter cells (28). Therefore, the lengthening of this division stage likely drives the increase in cell size observed in the mutant, while in a small number of cases (Fig. S2), synthesis of new septum is initiated before daughter cell separation is completed.

Despite several attempts, these phenotypes could not be complemented. However, as *mspA* is not part of an operon and in a previous work where the inactivation of genes to either side of *mspA* did not affect any MspA-associated phenotypes (27), we do not believe the transposon insertion is having any polar effects. To ensure that mutations that may have arisen during the transposon mutational process are not contributing to the effects described here, we used a back-crossed strain, where the transposon insertion was moved from the original mutation strain (JE2) into a new strain (SH1000). The effect on cell size and separation was common to the *mspA::*tn mutation in both strain backgrounds (Fig. 1A; Fig. S1). To further confirm this, we have also sequenced the genome of the wild type and mutant and found no mutations in any genes associated with the phenotypes described here (accession number: PRJNA1123932). As such, and as our data described later affirm, we are confident that the loss of expression of *mspA* is directly responsible for the effects on cell size and separation of the *mspA* mutants.

### Inactivation of MspA causes increased synthesis of lipoteichoic acid

**Fig 2:**
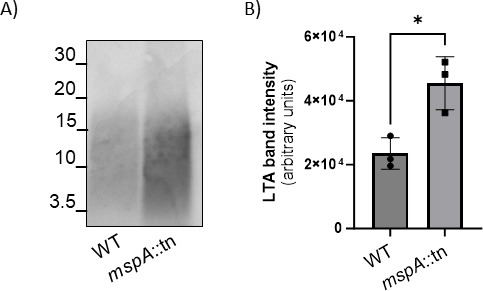
There is a greater abundance of LTA in the MspA-deficient cells. (**A**) Western blot with anti-LTA antibody on samples from SH1000 wild type (WT) and SH1000 *mspA*::tn (*mspA*::tn). (**B**) Quantification of the intensity of LTA Western blot bands performed with ImageJ (*n* = 3, *t*-test). *P = 0.017.

The inactivation of proteins involved in cell division or cell wall synthesis frequently causes an increase in cell size (29–32). For example, deletion mutants of the genes encoding for UgtP or LtaA, proteins responsible for the lipoteichoic acid (LTA) glycolipid anchor synthesis and flipping (Gründling & Schneewind, 2007a) display longer LTA structures, enlargement of cell size, defects in septa formation and higher susceptibility to lytic enzymes (33). Given the overlap between these phenotypes and those of the MspA-deficient cells described above, and the fact that LTA is anchored to the membrane and spans the membrane-proximal region of the cell wall (34, 35), we hypothesized that the loss of MspA may affect LTA biosynthesis. To test this, we first examined whether lipoteichoic acid abundance was altered in MspA-deficient cells. Western blots using anti-LTA antibodies (Fig. 2A; Fig. S3) and their quantification (Fig. 2B) showed that MspA-deficient cells grown overnight had significantly increased LTA content compared to the wild type. Based on their migration, LTA polymers appeared to be of similar molecular weight in both strains and did not display any clear differences in length.

### The increase in cell size of the MspA-deficient cells is associated with increased abundance of LTA

**Fig 3:**
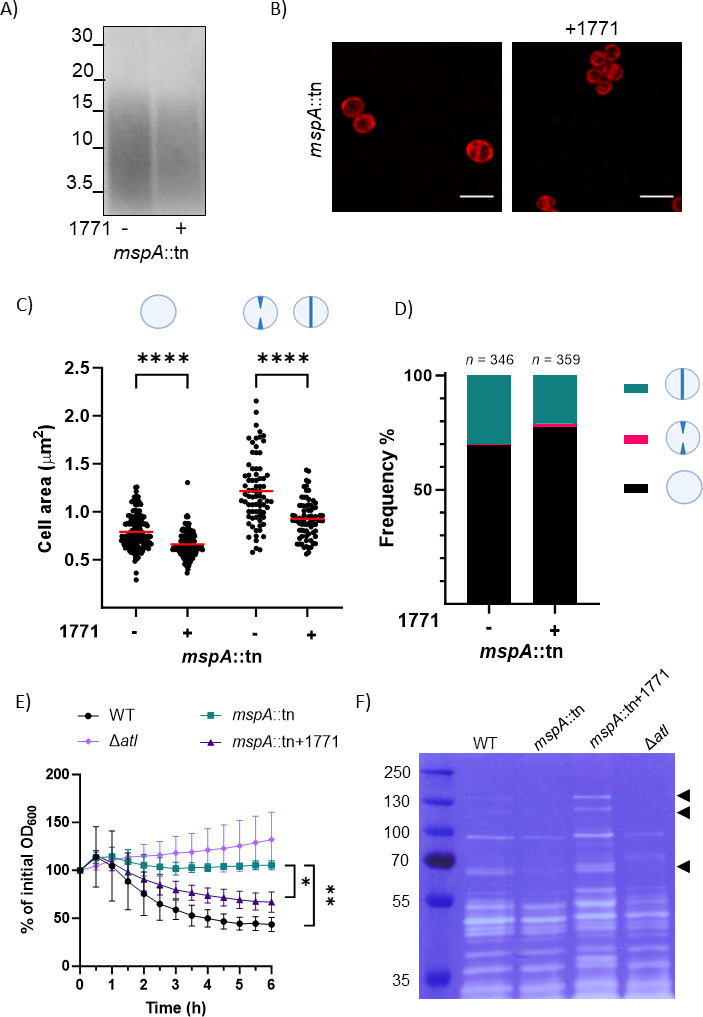
The increased abundance of LTA in the MspA-deficient cells alters cell size and cell cycle progression. (**A**) Western blot with anti-LTA antibody on samples from SH1000 *mspA*::tn grown in the presence or absence of 2 µg/mL of compound 1771. (**B**) Sample confocal fluorescent microscopy images of fixed *mspA*::tn cells grown in the presence or absence of compound 1771 (2 µg/mL) and stained with Nile Red membrane dye. Scale bar: 2 µm. (**C**) Quantification of cell area of Nile Red-stained cells grown in the presence or absence of compound 1771 (2 µg/mL). In non-dividing cells (left) and dividing cells (right), 1771 significantly decreases the area of MspA-deficient cells. Red lines indicate the mean. Data were analyzed with two-way analysis of variance (ANOVA) with Sidak’s post hoc test (non-dividing cells: *n* = 168, dividing cells: *n* = 76). (**D**) Frequency of MspA-deficient cells stained with Nile Red in each of the division stages when grown in the presence or absence of compound 1771 (2 µg/mL). The 1771 treatment reduces by ~30% the proportion of cells with a fully formed septum that have not separated yet. The total number of cells analyzed is indicated on top of the bars. (**E**) Triton X-100 triggered lysis of the SH1000 wild type and the SH1000 *mspA*::tn strain grown in the presence or absence of compound 1771 (2 µg/mL); the SH1000 Δ*atl* mutant has been provided as a control. Lysis is measured by monitoring OD_600_ over 6 h after exposure to Triton X-100 (*n* = 3, two-way ANOVA with Tukey’s post hoc test; at 6 h; WT vs *mspA*::tn; *mspA*::tn vs *mspA*::tn 1771). (**F**) Zymogram of cell wall fractions extracted from the wild type and the *mspA* mutant grown in the presence or absence of compound 1771 (2 µg/mL) and SH1000 Δ*atl*. Cell wall extracts were run on an SDS-PAGE with embedded heat-killed *S. aureus*. The peptidoglycan hydrolases were renatured, and their activity was visualized by methylene blue staining. Black arrowheads indicate zones of clearance corresponding to Atl’s activity. *P = 0.04, **P = 0.002, ****P < 0.0001.

To test whether the increase in size in the MspA-deficient cells was due to the altered amount of LTA, we cultured the MspA-deficient strain overnight with compound 1771. This has been previously shown to decrease LTA biosynthesis (36, 37), although the target of inhibition is yet unclear (37, 38). Since LTA is essential, we chose a sub-MIC of the compound which did not affect CFU per milliliter counts after overnight growth (Fig. S4) and reduced the amount of LTA produced (Fig. 3A; Fig. S5). Using Nile Red dye for membrane staining and confocal microscopy, we found that the 1771 treatment did not affect cell integrity, and there was no lysis observed (Fig. 3B). Measurements of the cell area of the Nile Red-stained mutant and wild-type cells grown in the absence of 1771 confirmed the enlarged cell phenotype of the MspA-deficient cells observed with TEM (Fig. S6). In both non-dividing and dividing cells, the 1771 treatment significantly decreased the size of MspA-deficient cells (Fig. 3C). This suggests that the greater abundance of LTA observed for the MspA-deficient cells is linked with the observed increase in cell size.

### The greater abundance of LTA in MspA-deficient cells affects the cell cycle by suppressing autolysis

Given the association of increased LTA abundance and cell size, we next tested whether the elevated LTA levels were also responsible for the higher proportion of mutant cells with a mature septum. We cultured the MspA-deficient cells with and without the 1771 LTA-inhibiting compound as above, stained them with Nile Red, and quantified the prevalence of cells in the different cell cycle stages. While cells with a fully formed septa were readily visible, we could identify a smaller proportion of cells with incomplete septa compared to TEM images. This difference is likely due to resolution limits of light microscopy and the fact that Nile Red stains only the membrane rather than the whole envelope. Compound 1771 reduced the proportion of MspA-deficient cells with mature septa by ~30%, from 30% to 21% (Fig. 3D).

Critical to cell division and growth is cell wall turnover, which for *S. aureus* is mediated by the activity of more than 15 peptidoglycan hydrolases. These enzymes, also known as autolysins, mediate daughter cell separation after septum formation (39). Both WTA and LTA and their D-alanylation have been implicated in controlling the activity and localization of autolysins (40–42). Therefore, we employed an autolysis assay to test whether autolytic activity was altered in the absence of MspA and if the increased amount of LTA could be responsible for it. A deletion mutant in the main autolysin *atl* was used as a control strain. Detergent-induced autolysis of the MspA-deficient cells was indeed significantly reduced compared to the wild type (Fig. 3E), suggesting a dysregulation of the activity of autolytic enzymes. Crucially, addition of compound 1771 to the medium restored the autolytic activity of the MspA-deficient cells (Fig. 3E). Next, zymography was used to assess the abundance and activity of hydrolases in the same strains. Matching the results from the autolysis assay, we found that the cell wall extracts from MspA-deficient cells showed fewer and fainter clearance bands in the zymogram compared to the wild type, suggesting reduced activity of peptidoglycan hydrolases. The bands became again readily visible upon addition of the 1771 compound (Fig. 3F). This suggests that the excess LTA causes dysregulation of the autolytic enzyme activity which, in turn, is likely responsible for the observed delay in autolytic splitting of daughter cells. Atl is proteolytically processed (43), explaining the absence of several bands in the cell wall extracts of the *atl* mutant compared to the isogenic wild type. In particular, two bands around the 130-kDa mark and two bands below the 70-kDa mark were absent in both the *atl* and the *mspA* mutant, suggesting that Atl’s abundance and activity specifically are reduced in the the MspA-deficient cells.

### Characterization of the MspA interactome

**Fig 4:**
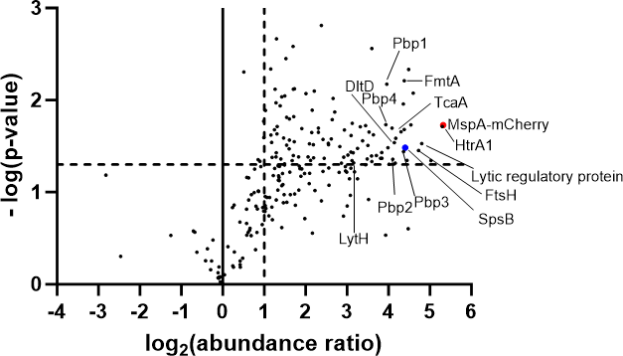
Proteins involved in envelope biogenesis are co-immunoprecipitated with MspA-mCherry. Volcano plot showing the abundance of proteins identified via liquid chromatography-mass spectrometry in the elution fractions of a co-immunoprecipitation experiment performed with MspA-mCherry as a bait. Proteins were considered as significantly enriched with the MspA-mCherry bait (in red) compared to the negative control if the log(*P* value) was >1.3 and the log_2_(fold change) >1. The significance thresholds are marked by dotted lines. Some of the significantly enriched proteins involved in cell envelope biogenesis are labeled on the graph; SpsB is highlighted in blue.

Our findings have established that without MspA, there is an increase in abundance of LTA which, in turn, deregulates autolytic activity important for daughter cell separation, a process that ultimately leads to an increased cell size. To understand in greater detail how MspA could affect LTA synthesis, we next sought to identify MspA’s binding partners. We employed co-immunoprecipitation (co-IP) using MspA tagged at the C-terminus with mCherry as bait as attempts to raise an antibody against MspA were unsuccessful. Mass spectrometry analyses were performed on the co-IP elution fractions, and the abundance of proteins co-eluted with MspA-mCherry was compared with the abundance of the same proteins in the negative control sample. Several membrane-anchored and cell wall proteins involved in envelope biogenesis were found to be significantly enriched with MspA-mCherry, including the four penicillin-binding proteins, the DltD protein responsible for d-alanylation of teichoic acids (44), FmtA that hydrolyzes the bond between D-alanine and teichoic acid backbones (45), the cell wall amidase LytH (31), and TagH involved in teichoic acid export (46) (Fig. 4; Table S1).

**Fig 5:**
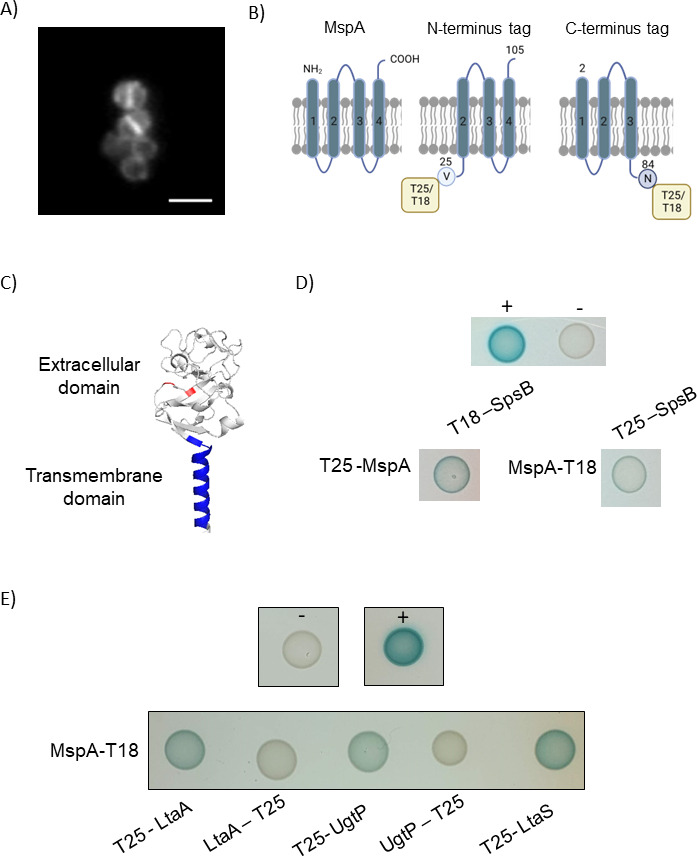
MspA interacts with the LTA synthesis enzymes. (A) Widefield fluorescent microscopy of SH1000 pOS1 MspA-mCherry showing localization of MspA at the cell membrane. Scale bar: 1 µm (B) Diagram of the MspA protein and of its truncated versions which were cloned in the bacterial two-hybrid plasmids to maintain the T18 or T25 tag intracellularly. Created with BioRender. (C) AlphaFold structure of the signal peptidase SpsB. The transmembrane domain is highlighted in blue (amino acids 8–28), and the active site residues in the extracellular domain are highlighted in red (amino acids 36 and 77). (D) Bacterial two-hybrid analysis between MspA tagged with the T25 subunit at the N-terminus and the T18 subunit at the C-terminus and full-length SpsB, tagged at the N-terminus, as indicated. MspA interacts SpsB. Representative of three biological replicates. pKT25 and pUT18 plasmids were co-transformed as negative control (−) and pKT25 – zip and pUT18C – zip as a positive control (+). (E) Bacterial two-hybrid analysis between MspA tagged with the T18 subunit at the C-terminus and LtaA, UgtP, and LtaS tagged with the T25 subunit at the N- or C-terminus, as indicated. LtaS was tested only with the tag at the N-terminus as the C-terminus localizes extracellularly. MspA interacts with LtaA, UgtP, and LtaS tagged at the N-terminus. Representative of three biological replicates. pKT25 and pUT18 plasmids were co-transformed as negative control (−) and pKT25 – zip and pUT18C – zip as a positive control (+).

Of particular note among the MspA interactome proteins was SpsB, a type I signal peptidase deemed to be essential (47) and a potential target for therapeutics (48, 49). This protein attracted our interest, given it association with LTA biosynthesis, where it cleaves LtaS between alanine 217 and serine 218 residues, separating the N-terminal five transmembrane helices from the extracellular catalytic domain of LtaS (eLtaS) (23) and releases eLtaS into the cell wall. This cleavage process has recently been shown to be coupled with LTA biosynthesis (24). Should MspA interact directly with SpsB, it could affect this cleavage activity and as a consequence affect LTA biosynthesis by LtaS. To verify the direct interaction between MspA and SpsB, we undertook a bacterial two-hybrid (BACTH) approach. Structural predictions suggest that MspA is a membrane protein. We first confirmed this by fusing the *mspA* coding sequence with mCherry and performing fluorescent widefield microscopy. This analysis showed that MspA indeed localizes in the membrane in a uniform pattern (Fig. 5A). The slightly more intense signal observed at the septum in dividing cells is expected due to the doubling of the membrane layers at that site. As both the N- and C- termini of MspA are predicted to be exposed in the extracellular space (Fig. 5B), a specific BACTH construct design was required. We cloned only the last three transmembrane domains in the constructs where MspA was fused with the T25 or T18 tag at the N-terminus, and only the first three transmembrane domains in the constructs where MspA was fused with the T25 or T18 tag at the C-terminus (Fig. 5B). This allowed the T25 or T18 tag to localize intracellularly and, if an interaction with another tagged protein took place, catalyze the BACTH-associated conversion of ATP to cAMP (50). SpsB has a transmembrane domain which anchors it to the cell, while the catalytic site is located in the extracellular domain (Fig. 5C). The full-length *spsB* gene and the extracellular domain only were cloned in the BACTH plasmids, and when combined with the MspA BACTH plasmid constructs, we found that MspA interacts with full-length SpsB but not with the extracellular domain (Fig. 5D; Fig. S7), suggesting that MspA associates with the membrane spanning domain of SpsB. We also examined whether MspA interacted with other LTA-associated membrane-bound enzymes that were not identified as part of the co-IP experiment and found that it also appears to bind to LtaA, LtaS, and UgtP (Fig. 5E; Fig. S8). As BACTH can produce false-positive results and the membrane-bound localization of all our enzymes of interest could confound matters, we also examined whether our BACTH experiment would suggest that MspA could bind flotillin (FloA), a membrane-bound protein responsible for the assembly and stability of functional membrane microdomains. These are domains in bacterial membranes similar to the eukaryotic lipid rafts formed in *S. aureus* by the co-localization of staphyloxanthin lipids and the scaffold protein FloA (51, 52). However, we did not observe any interactions between MspA and FloA using this approach (Fig. S9), supporting the validity of our positive BACTH results.

### MspA interaction with SpsB alters LtaS processing

**Fig 6:**
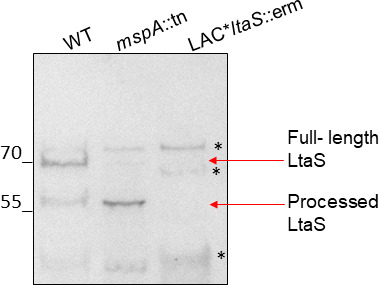
MspA-deficient cells display alterations in LtaS processing. Western blot with anti-LtaS antibody on cell fractions extracted from SH1000 (WT) and SH1000 *mspA*::tn (*mspA*::tn) and LAC* *ltaS*::erm, an *ltaS* knock-out suppressor strain. Bands deemed as non-specific as they were detected in the *ltaS* knock-out strain are indicated with an asterisk (*). The bands corresponding to full-length LtaS (70 kDa) and the eLtaS processed extracellular domain (55 kDa) are indicated with red arrows.

Having demonstrated by two independent means that MspA interacts with SpsB, we sought to determine whether this association was functional and affected LtaS processing in the *mspA* mutant. An anti-LtaS antibody recognizing the extracellular domain of LtaS (23) was used to assess the relative abundance of the full-length protein and cleaved eLtaS catalytic domain. A striking alteration in LtaS processing was observed in the *mspA* mutant. While in the wild type most of the LtaS protein was found in the full-length form (70 kDa) and a smaller amount of processed eLtaS (55 kDa) was detected, the opposite was observed in the *mspA* mutant, where most of the LtaS was detected in the processed form and the full form of the enzyme was barely detected (Fig. 6C; Fig. S10). This suggests that LtaS processing is increased in the *mspA* mutant, resulting in increased eLtaS catalytic domain being associated with the cell (23, 24). We suggest that an increased processing of LtaS to eLtaS in the *mspA* mutant increases the release of the catalytic domain, which in turn causes the observed increase in LTA synthesis. These data suggest that MspA affects LtaS processing via directly interacting and blocking SpsB activity, which as a result is increased in the *mspA* mutant.

## DISCUSSION

**Fig 7:**
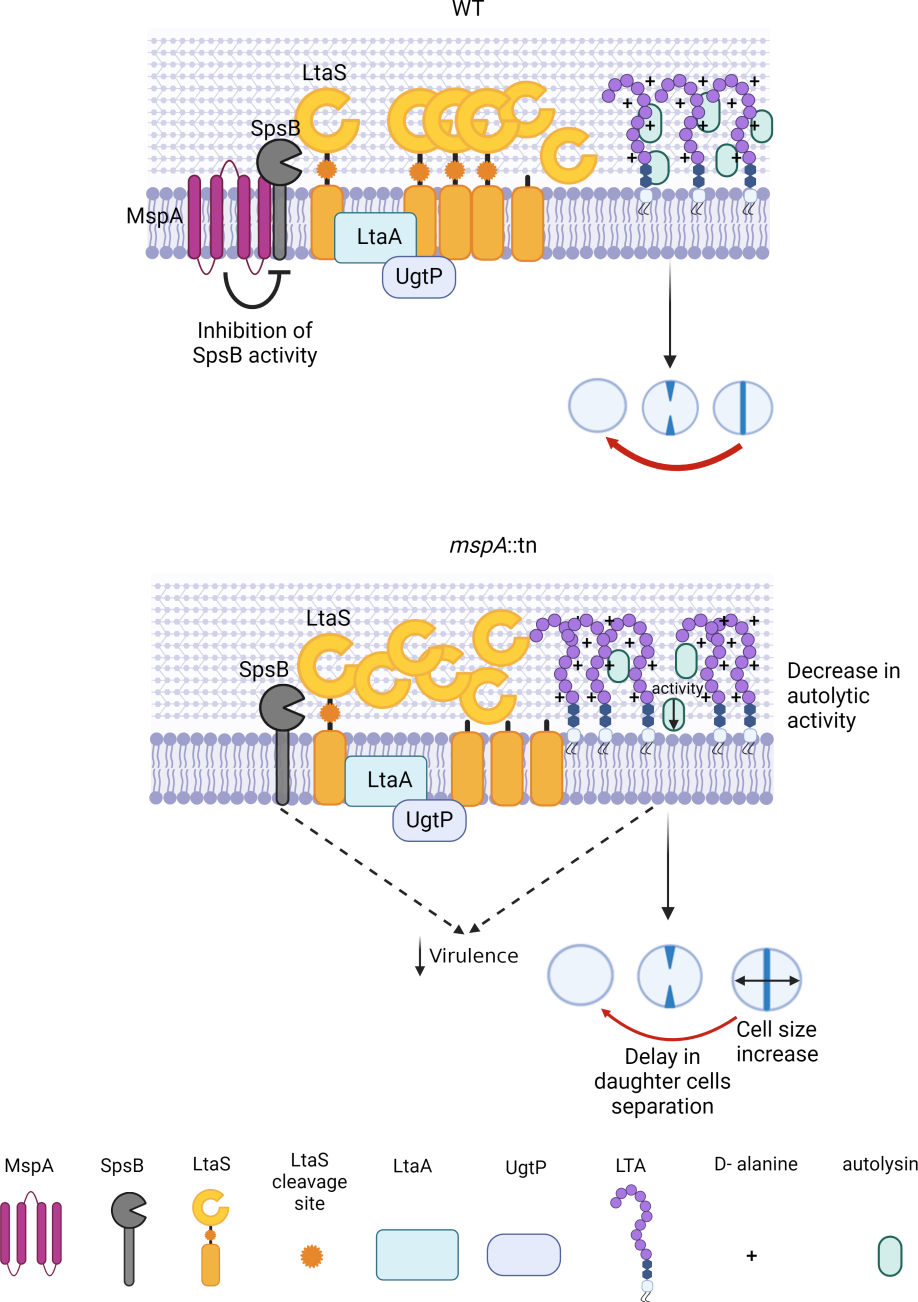
MspA negatively regulates LTA production by interacting with SpsB and inhibiting its activity. MspA maintains normal cell cycling and cell size by regulating LtaS processing, LTA synthesis and, indirectly, autolysis activity. The LTA defects observed in the absence of MspA as well as altered SpsB activity could also underpin the reduced virulence.

We have previously shown that the MspA protein is critical to the virulence of *S. aureus* due to the effect it has on toxin production, membrane stability, staphyloxanthin biosynthesis, iron homeostasis, and susceptibility to components of the innate immune system. In this study, we decipher the molecular basis of this cellular role. We show that MspA directly interacts with the signal peptidase SpsB, which cleaves LtaS, altering its activity and LtaS processing. This contributes to LTA homeostasis, which, in the absence of MspA, leads to an increase in cell size, likely due to a delay in autolytic daughter cell separation. A graphical summary of this process is provided (Fig. 7). We also showed an interaction between MspA and the LTA synthetic enzymes UgtP, LtaA and LtaS. However, since such interaction was supported only by a single method, we do not consider it to be the primary mechanism whereby MspA controls LTA synthesis.

Autolytic enzymes affect both cell morphology and the cell cycle progression. In *S. aureus*, deletion of Atl and Sle1 autolysins leads to elongation of the cell cycle (28), while Sle1 inactivation also causes a delay in daughter cell separation, resulting in an increase in cell size similarity to what is observed in the absence of MspA (28, 53, 54). An *atl* deletion mutant, in contrast, exhibits initiation of new septa before the previous round of cell division is completed, resulting in disturbed cell separation and cell clumping (43, 55, 56). Similar formation of premature septa was also observed in a small number of cells deficient for MspA. In contrast to *sle1*, deletion of *atl* does not alter the cell size (57). However, upregulation of secondary hydrolases in the absence of Atl has been suggested to compensate for defects caused by *atl* deletion (58, 59). LTA is known to regulate the autolytic process, although the detailed mechanisms are not well understood. LTA has previously been shown to bind the Atl amidase repeats, likely targeting Atl to the septum and modulating its activity, as increasing concentration of LTA decreases the peptidoglycan amidase activity (42). Accordingly, other mutants with altered LTA synthesis display defects in autolysis. *ugtP* deletion mutants were shown to have reduced autolytic activity (60), and *ltaS* deletion strains with suppressor mutations were reported to have decreased abundance of autolysins in the cell wall (17, 20). Lack of UgtP and LtaA leads to longer LTA polymers, which, similar to lack of MspA, lead to an increased cell size due to defects in cell cycle progression. In the case of UgtP and LtaA, however, septum formation rather daughter cell separation is delayed (61). This shows that LTA abundance and length can affect the cell cycle at different stages.

We provide evidence that MspA contributes to maintaining a homeostatic level of LTA in the cell wall. Recently, *ltaS* has been found to be transcriptionally downregulated in stationary phase by the essential two-component system WalKR (62), confirming the importance of controlling and balancing the polymer synthesis. Interestingly, when first characterized, the SpsB signal peptidase was thought to cleave LtaS to inactivate the LTA synthase (23). However, a recent study has shown that the cleaving LtaS to eLtaS and the release of the catalytic domain in the cell wall is a process coupled to LTA synthesis, rather than finalized to inhibit it (24). Our findings support this updated model, since increased LTA synthesis in the MspA-deficient cells is associated with an increased amount of processed eLtaS relative to the full-length enzyme. Ibrahim et al. (24) observed that LtaS processing via SpsB is triggered by LtaS interaction with the glycolipid anchor Glc_2_DAG, synthesized and flipped by the enzymes UgtP and LtaA. As a result, *ugtP* and *ltaA* mutants display reduced eLtaS release (24). Here we describe an additional mechanism of regulation of SpsB activity via MspA. Although it is possible that LtaS processing is altered in the MspA-deficient cells due to changes in lipid composition and Glc_2_DAG abundance in particular, we believe that the physical interaction between MspA and SpsB points toward a direct regulation of the signal peptidase’s activity by our protein of interest. Further work is needed to further elucidate the molecular details of this inhibitory dynamic.

The synthesis and turnover of cell envelope components are known to impact virulence and pathogenicity of *S. aureus*. For instance, the membrane lipid lysyl-phosphatidylglycerol, LTA (63), and WTA (64) control sorting and secretion of toxins, and deletion of the scaffold protein FloA causes a decrease in virulence as it is needed to stabilize RNase Y, which degrades small RNAs downregulating toxins (65). Additionally, LTA has been showed to impact virulence, as *ltaA* and *ugtP* deletion mutants have attenuated pathogenicity (22, 66). It is therefore possible that the increased abundance of LTA in the absence of MspA causes major structural changes of the membrane that has pleiotropic effects on virulence. Alternatively, alteration in SpsB activity in the *mspA* mutant could also account for defects in virulence. Chemical inhibition of SpsB followed by proteomic analyses of the secretome has suggested that SpsB’s signal peptidase activity is responsible for the secretion of several virulence factors, such as α-, γ-, and δ-hemolysins, proteases, immunoglobulin G-binding protein (Sbi), and lipases (67). Additionally, genetic inactivation of *spsB* has been shown to reduce cell wall deposition of adhesins and immunevasins [fibronectin-binding protein A, clumping factor A (ClfA) and IsdA], and to reduce infectivity *in vivo* (48). Therefore, the interaction between SpsB and MspA and alteration of SpsB activity in the *mspA* mutant could also explain the far-reaching impacts on virulence factor production that we have previously described in the MspA-deficient strain.

In conclusion, we have shown that MspA is a membrane protein that affects LtaS processing by SpsB, leading to a dysregulation of LTA biosynthesis with major pleiotropic consequences for the ability of the bacteria to cause disease. This work therefore describes a new link between envelope synthesis and *S. aureus* virulence and suggests that MspA could represent a promising target for future therapeutic development.

## MATERIALS AND METHODS

### Strains and culture conditions

*Staphylococcus aureus* strains used in this study are listed in Table 1. They were grown in tryptic soy broth at 37°C with shaking at 180 rpm unless otherwise specified. Chloramphenicol (10 µg/mL), kanamycin (50 µg/mL), and anhydrous tetracycline (200 ng/mL) were added where appropriate. *Escherichia coli* strains were grown in LB, Mach1, and DH5α cloning strains at 37°C with shaking at 180 rpm and in BTH101 at 30°C with shaking at 180 rpm. When appropriate, ampicillin (100 µg/mL) or kanamycin (30 µg/mL) was added.

**TABLE 1:** Strains used in this study

Strain name	Description	Reference
*Staphylococcus aureus*		
SH1000	Laboratory strain, 8325–4 with a repaired *rsbU* gene; SigB positive	(68)
SH1000 *mspA*::tn	*mspA* transposon mutant in SH1000	(27)
SH1000 pOS1 mspA-mCherry	SH1000 expressing *mspA* gene fused with mCherry on the pOS1 plasmid	This study
RN4220	restriction-negative derivative of NCTC8325-4	(69)
SH1000 pCN34	SH1000 carrying the empty pCN34 plasmid	This study
SH1000 pCN34 p*itet mspA-mCherry*	SH1000 expressing mspA fused with mCherry under the anhydrous tetracycline promoter on the pCN34 plasmid	This study
LAC*ltaS:erm suppressor US3 pass 4 (US3) (ANG2434)	*ltaS* deletion mutant suppressor with *gdpP* mutation, pass 4 (short: US3)	(20)
*Escherichia coli*		
Mach1	Cloning strain	Laboratory collection
DH5α	Cloning strain	Laboratory collection
BTH101	BACTH Δ*cya* strain; StrepR	(70)

### Genetic manipulations

Phusion polymerase (Thermo Fisher Scientific) was used for PCR amplification (oligonucleotide primers used in this study are listed in Table 2). New England Biolabs enzymes were used for restriction digestion and for ligation. The plasmids used and constructed in this study are listed in Table 3.

**TABLE 2:** Primers used in this study

Primer name	Sequence (5’ – 3’)
B2H mspA 25–105 F	ATAT**GGATCC**Cgttgtatttactcgcattttgag
B2H mspA 25–105 R	ATATGGTACCCGGAATAATACGATGGTTAAGATGAAATATATG
B2H mspA 2–84 F	ATATGGATCCCCaattttatctgattttactagcaatac
B2H mspA 2–84 R	ATATGGTACCCGGTTTACGCCTTTTAGATCTTTTTTAAG
B2H FloA F	ATAT**ggatcc**ctttagtttaagttttatcgtaatagc
B2H FloA R	ATATggtacccgatgttcaggtgactcatc
B2H SpsB Full F	ATATTCTAGAgttgaaaaaagaaatattggaatgg
B2H SpsB extracellular domain F	ATATTCTAGAggatccaactttgaaagatgg
B2H SpsB R	ATATGGTACCCGatttttagtattttcaggattgaaattatg
BTH_F	CAATTTCACACAGGAAACAGCTATGAC
pKT25/NT25_R	CGCCTTCTTGACGAGTTCTTCTG
pUT18/18C_R	GAAAAATAAACAAATAGGGGTTCCGCG
mspA_fusion_F	ATATggatccTTAATGCGCTTCAAAAAGTAAC
mspA_fusion_R	gacaccatGAATAATACGATGGTTAAGATGAAATATATG
mcherry_translational _fusion_F	CCATCGTATTATTCatggtgtcaaaaggtgaag
mcherry_translational _fusion_R	cttggCTGCAGttatttgtataattc
MspAmCherry F	ATATggtaccACCCTTTGAAACGGAGAG
MspAmCherry R	ATATgaattcttatttgtataattcatccataccacc

**TABLE 3:** Plasmids used in this study

Plasmid name	Description	Reference
pOS1 *mspA-mCherry*	pOS1 expressing *mspA* fused with mCherry under *mspA* native promoter	This study
pRMC2 *mspA-mCherry*	pRMC2 expressing *mspA* fused with mCherry under the anhydrous tetracycline inducible promoter	This study
pCN34	*S. aureus* shuttle vector	(71)
pCN34 pitet MspA-mCherry	pCN34 expressing *mspA* fused with mCherry under the anhydrous tetracycline inducible promoter	This study
pKT25	BACTH vector containing an isopropyl β-D-1-thiogalactopyranoside (IPTG) inducible promoter, T25, and multiple cloning site, KanR	(70)
pKNT25	BACTH vector containing an IPTG inducible promoter, multiple cloning site, and T25; KanR	(70)
pUT18	BACTH vector containing an IPTG inducible promoter, multiple cloning site, and T18; AmpR	(70)
pUT18C	BACTH vector containing an IPTG inducible promoter, T18, and multiple cloning site; AmpR	(70)
pKT25-zip	T25 fused to N-terminus of the leucine zipper of GCN4, KanR	(70)
pUT18C-zip	T18 fused to N-terminus of the leucine zipper of GCN4; AmpR	(70)
pKT25-*mspA*	MspA amino acids 25–105 with T25 tag fused at the N-terminus	This study
pKNT25-*mspA*	MspA amino acids 2–84 with T25 tag fused at the C-terminus	This study
pUT18-*mspA*	MspA amino acids 2–84 with T18 tag fused at the C-terminus	This study
pUT18C-*mspA*	MspA amino acids 25–105 with T18 tag fused at the N-terminus	This study
pKT25-*ltaA*	T25 fused to N-terminus of LtaA	(34)
pKT25-*ltaS*	T25 fused to N-terminus of LtaS	(34)
pKT25-*ugtP*	T25 fused to N-terminus of UgtP	(34)
pKNT25-*ltaA*	T25 fused to C-terminus of LtaA	(34)
pKNT25-*ugtP*	T25 fused to C-terminus of UgtP	(34)
pUT18-*ltaA*	T18 fused to C-terminus of LtaA	(34)
pUT18- *ugtP*	T18 fused to C-terminus of UgtP	(34)
pUT18C-*ltaA*	T18 fused to N-terminus of LtaA	(34)
pUT18C-*ltaS*	T18 fused to N-terminus of LtaS	(34)
pUT18C-*ypfP*	T18 fused to N-terminus of UgtP	(34)
pKT25-*floA*	T25 fused to N-terminus of FloA	This study
pKNT25-*floA*	T25 fused to C-terminus of FloA	This study
pUT18-*floA*	T18 fused to C-terminus of FloA	This study
pUT18C-*floA*	T18 fused to N-terminus of FloA	This study
pKT25-*spsB* full	Full SpsB sequence with T25 fused to N-terminus	This study
pKNT25-*spsB* full	Full SpsB sequence with T25 fused to C-terminus	This study
pUT18-*spsB* full	Full SpsB sequence with T18 fused to C-terminus	This study
pUT18C-*spsB* full	Full SpsB sequence with T18 fused to N-terminus	This study
pKT25-*spsB*Δ	Extracellular SpsB domain (amino acids 38–191) with T25 fused to N-terminus	This study
pKNT25-*spsB* full	Extracellular SpsB domain (amino acids 38–191) with T25 fused to C-terminus	This study
pUT18-*spsB* full	Extracellular SpsB domain (amino acids 38–191) with T18 fused to C-terminus	This study
pUT18C-*spsB* full	Extracellular SpsB domain (amino acids 38–191) with T18 fused to N-terminus	This study

### Electron microscopy

Strains were cultured at 37°C in 5-mL TSB in a 50-mL falcon tube for 18 h. An 800-µL volume was removed and pelleted. Cells were fixed by resuspending pellets in 2.5% glutaraldehyde in cacodylate buffer (pH 7.3) and stored at 4°C until further processing. Fixed pellets were resuspended in BSA/glutaraldehyde gel at 10°C–20°C and once again pelleted. Pellets were postfixed with osmium ferrocyanide/osmium tetroxide in cacodylate buffer and stained with 2% uranyl acetate and Walton’s lead aspartate. Finally, samples were subject to ethanol dehydration then infiltration with propylene oxide and EPON resin mix. Embedded blocks were polymerized for 48 h at 60°C. Multiple 70-nm sections were cut on a Leica UC7 ultramicrotome and imaged using a FEI Tecnai T12 microscope at the Wolfson Bioimaging Facility at the University of Bristol.

### Analysis of LTA and LtaS via Western blot

Strains were grown overnight and ~10^9^ CFU/mL bacteria were transferred to a 2-mL Lysing matrix B tube (MP Biomedicals) and bead-beaten for 1 minute at 5 m/s twice. Beads were settled via centrifugation at 2,000 rpm in a tabletop centrifuge, and 500 µL of supernatant was centrifuged at 13,000 rpm for 15 minutes. The supernatant was discarded and the pellet was resuspended in 100 µL of a 1:1 solution of 100-mM Tris-HCl, pH 7.4, with EDTA-free protease inhibitor cocktail (one tablet in 10 mL of buffer, Roche) and 4× NuPage LDS sample buffer (Invitrogen). The resuspended pellets were boiled for 20 minutes and centrifuged at 13,000 rpm for 5 minutes. The supernatant was harvested and stored at −20°C. Samples were heated at 70°C for 10 minutes and loaded on FastGene PAGE Gel, 4%–12% (GeneFlow). The gels were run in 2-morpholinoethanesulphonic acid (MES) buffer (NuPage) and transferred to methanol-activated polyvinylidene fluoride (PVDF) 0.2-µm membrane (Cytiva) in a semi-dry transfer at 25 V for 30 minutes with transfer buffer (600-mM Tris, 600-mM glycine, 280-mM tricine, 0.05% SDS, and 2.5-mM EDTA). Membranes were blocked with 3% BSA in phosphate-buffered saline (PBS), stained with 1:1,000 α-LTA mAb 55 (Hycult BioTech) and with 1:10,000 anti-mouse IgG peroxidase HRP (Sigma-Aldrich) or with 1:5,000 α-eLtaS polyclonal serum and 1:10,000 anti-rabbit IgG peroxidase HRP (Sigma-Aldrich). IgG from human serum (50 µg/mL, Merck) in PBS was added to all blocking and antibody incubations. Membranes were developed with Metal Enhanced DAB Substrate Kit (Thermo Fisher Scientific). Blot images were taken with Genesys acquisition system (Syngene), and band intensity was quantified with ImageJ.

### Fluorescence microscopy

For imaging of membrane-stained *S. aureus*, 1 mL of bacteria grown overnight with or without 2-µg/mL compound 1771 was washed three times in PBS and resuspended in 1-mL PBS with 10-µg/mL Nile red. Samples were incubated at 37°C in the dark for 5 minutes, washed three times in PBS, resuspended in 4% paraformaldehyde in PBS, incubated at room temperature for 15–30 minutes, and stored at 4°C in the dark. For image acquisition, 2 µL of sample was mounted on a slide with 1.2% agarose and covered with a coverslip. Images were taken with a with Leica SP8 AOBS confocal laser scanning microscope attached to a Leica DM I8 inverted epifluorescence microscope. Images were analyzed with ImageJ Fiji (72). For the analysis of MspA localization, the *mspA-mCherry* fusion was generated, amplifying the *mspA* from JE2 genomic DNA with primers mspA_fusion_F and mspA_fusion_F and mCherry from the pOS1-mCherry plasmid with primers mcherry_translational _fusion_F and mcherry_translational _fusion_R. The two PCR products were fused in a third PCR with primers mspA_fusion_F and mcherry_translational _fusion_R. This PCR product was digested with *Bam*HI and *Pst*I and cloned into pOS1 digested with the same enzymes. SH1000 cells carrying the expression plasmid pOS1 mspA-mCherry were grown overnight in TSB, followed by 1:100 dilution in fresh medium and incubation at 30°C upon shaking. Mid-logarithmic growth phase culture (0.5 µL) was transferred to 1.2% agarose-H_2_O slides followed by microscopy. The imaging was carried out with Nikon Eclipse Ti-E microscope equipped with Nikon CFI APO TIRF ×100/1.49 objective, Cobolt Jive 100 561-nm solid-state laser light source and Andor iXon Ultra 897 EMCCD camera. Images were acquired with Nikon NIS Elements AR 5.11 and processed with Fiji using PureDenoise algorithm (72, 73).

### Autolysis assay

Strains were grown overnight and diluted to an OD_600_ of 0.05 in 5 mL of TSB medium or TSB medium with or without 2 µg/mL of compound 1771 for the *mspA*::tn mutant. They were then grown to an OD_600_ of ~0.3 to 0.5 and washed in ice-cold water once. They were normalized to an OD_600_ of 1 in water with 0.1% Triton X-100. Two hundred microliters of culture in triplicate was transferred to a flat-bottom 96-well plate, and the OD_600_ was monitored over 6 h every 30 minutes with shaking at 500 rpm before each reading with SPECTROstar Nano plate reader.

### Zymography

Strains were grown overnight and diluted to an OD_600_ of 0.05 in 5 mL of TSB medium or TSB medium with or without 2 µg/mL of compound 1771 for the *mspA*::tn mutant. Once they reached an OD_600_ of ~0.3 to 0.5, 1 mL of culture was harvested by centrifugation (13,000 rpm for 1 minute), and the pellet was resuspended in 100-µL Tris-HCl, pH 7.5, supplemented with 20% sucrose and 10-mM MgCl_2_. Ten microliters of lysostaphin (1 mg/mL) was added, and samples were incubated at 37°C with shaking at 180 rpm to digest the cell walls. Samples were then centrifuged at 8,000 rpm for 3 minutes to pellet the protoplasts. Fifty microliters of supernatant was mixed with 50 µL of 2× SDS loading buffer. Twenty microliters of this sample was loaded on a 10% SDS PAGE made with an overnight SH1000 culture resuspended in water and autoclaved in place of water. Gels were run in Tris-glycine running buffer (0.025-M Tris, 0.192-M glycine, 0.1% SDS, pH 8.5), washed three times in water, and incubated statically overnight at 37°C in renaturation buffer (50-mM Tris-HCl, pH 7.5, 0.1% Triton X-100, 10-mM CaCl_2_, and 10-mM MgCl_2_). Gels were then stained with a solution of 0.1% methylene blue 0.01% KOH and destained with multiple washes in water to visualize bands of clearance.

### Co-immunoprecipitation

The *mspA-mCherry* fusion was amplified from pOS1 mspA-mCherry with primers MspAmCherry F and MspAmCherry F. The PCR product was digested with enzymes *Kpn*I and *Sal*I and cloned into pRMC2. pRMC2 MspA-mCherry was digested with enzymes *EcoR*I and *Sal*I to clone MspA-mCherry and the p*itet* promoter in pCN34, cut with the same enzymes. SH1000 pCN34 and SH1000 pCN34 p*itet* MspA-mCherry were cultured overnight. Five undred microliters of overnight culture was used to inoculate 50 mL of TSB supplemented with kanamycin and anhydrous tetracycline to induce expression of the fusion protein. The culture was grown to exponential phase (OD_600_ 0.5), centrifuged, and resuspended in 500 µL of co-IP buffer (one tablet of protease inhibitor dissolved in 7 mL of 50-mM Tris/Cl, pH 7.5, 150-mM NaCl, 20-mM MgCl, and 0.2% Tween 20). The resuspension was transferred to a 2-mL Lysing matrix B tube (MP Biomedicals) and bead-beaten for 1 minute at 5 m/s twice. The lysing tubes were centrifuged at 2,000 rpm for 2 minutes to settle the beads, and the remaining lysate was centrifuged at 13,000 rpm for 5 minutes. Two hundred microliters of the supernatant was diluted with 300 µL of co-IP buffer and mixed with 25 µL of ChromoTek RFP-Trap Magnetic Agarose beads (Chromotek) previously equilibrated with 500 µL of co-IP buffer. The lysate was mixed with the beads rotating end over end at 4°C overnight. The beads were washed three times with 500 µL of wash buffer (50-mM Tris/Cl, pH 7.5, and 150-mM of NaCl) and transferred to a clean tube. Eighty microliters of 2× SDS loading buffer was added to the tube and bound proteins by boiling at 95°C for 5 minutes. The eluted sample was analyzed by liquid chromatography-mass spectrometry. The experiment was repeated in biological triplicate.

### Bacterial two-hybrid

Genes were amplified from *S. aureus* JE2 genomic DNA with primers listed in Table S3. Amplification products and pKT25, pKNT25, pUT18, and pUT18C plasmids were digested with *BamH*I and *Kpn*I enzymes and ligated. Two microliters of ligation mixture was transformed into Mach1 or DH5α competent cells and screened for insertion with primers BTH_F and pKT25/NT25_R or pUT18/18C_R. One positive clone per construct was grown overnight for plasmid extraction. Correct insertion was checked, amplifying the construct with BTH_F_primer and reverse primer matching the insert. One plasmid per gene was also sequenced. Pairwise combinations of plasmids were co-transformed in BTH101 *E. coli* cells. Three colonies per co-transformation were grown overnight with appropriate antibiotics, and 1 mM of IPTG and 10 µL per replicate were dotted on LB containing antibiotics, 50 µg/mL of X-gal, and 1-mM IPTG. Plates were incubated at 30°C for 24 h.

### Statistical analyses

Statistical analyses indicated in the figure legends were performed using GraphPad Prism version 9.2.

## SUPPLEMENTAL MATERIAL

Supplemental material mbio.01512-24-s0001.docx Figures S1 to S10; Table S1.

ASM does not own the copyrights to Supplemental Material that may be linked to, or accessed through, an article. The authors have granted ASM a non-exclusive, world-wide license to publish the Supplemental Material files. Please contact the corresponding author directly for reuse.
