# Supplementary material for: Lipoteichoic acid biosynthesis by Staphylococcus aureus is controlled by the MspA protein
Source: mBio. 2024 Jul 22;15(8):e01512-24. doi: 10.1128/mbio.01512-24 (PMC11323550; doi:10.1128/mbio.01512-24)
Supplement: Supplemental material — Figures S1 to S10; Table S1. [file mbio.01512-24-s0001.docx]

**Supplementary Figures**


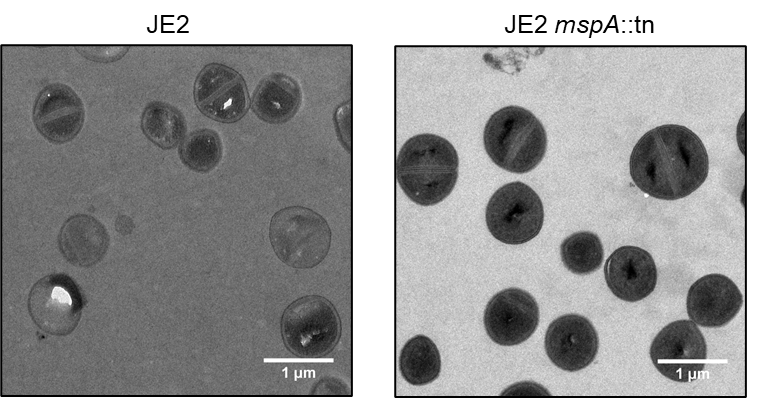


**Figure S1. MspA inactivation in the JE2 background causes an increase in cell size.** Transmission electron microscopy micrographs of JE2 wild-type and isogenic *mspA* transposon mutant cells.


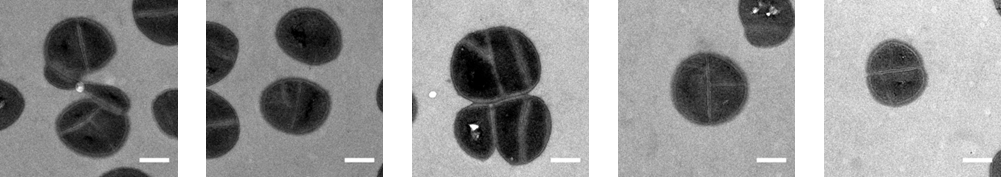


**Figure S2. TEM images of SH1000 *mspA*::tn mutant cells with irregular septa.** A small number of SH1000 *mspA*::tn cells have multiple septa for cells and/or septa that are not perpendicular to the previous septal plane. These defects were not observed in SH1000 wild-type. Scale bar: 0.5 µm.


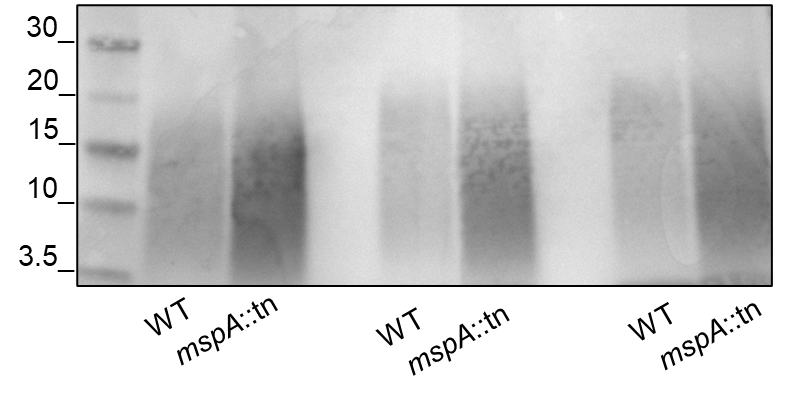


**Figure S3. The MspA-deficient cells displays increased LTA abundance.** Western blot with anti – LTA antibodies on cell samples from SH1000 and SH1000 *mspA*::tn.

**Figure S4. Viability of the MspA-deficient cells is not affected by addition of 2 µg/ml of 1771 compound**. CFU/ml counts of the SH1000 *mspA*::tn mutant after overnight growth in the presence of absence of 2 µg/ml of 1771.


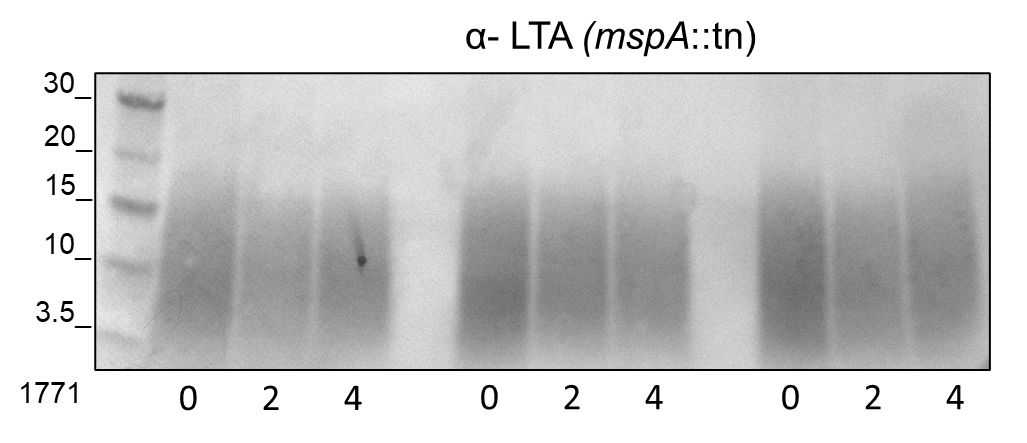


**Figure S5.** **Treatment with the 1771 compound reduces LTA synthesis.** Western blot with anti-LTA antibody on samples of MspA-deficient cells grown with 0, 2 or 4 µg/ml of 1771 compound.


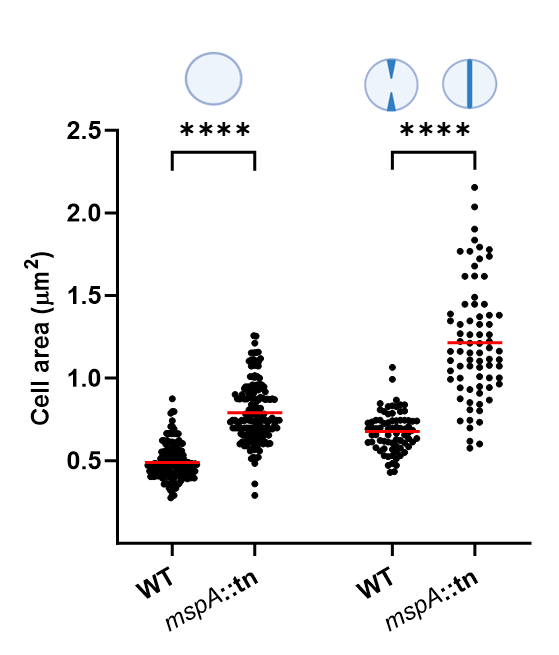


**Figure S6. Quantification of cell area of SH1000 (WT) and SH1000 *mspA*::tn (*mspA*::tn) cells stained with Nile Red and imaged with fluorescent microscopy.** Non dividing (left) and dividing (right) MspA-deficient cells have a significantly increased area compared to wild-type cells. Data were analysed with a two-way ANOVA with Sidak’s *post-hoc* test. Non-dividing cells: n= 168, ****, P<0.0001. Dividing cells: n= 76, ****, P<0.0001.


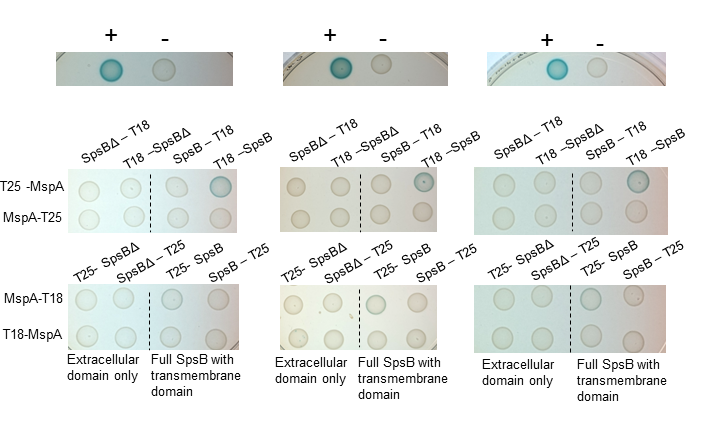


**Figure S7. All bacterial two-hybrid interactions tested between MspA and SpsB.** MspA does not interact with the extracellular domain of SpsB (SpsBΔ, on the left) and it interacts with the full-length SpsB protein (on the right). In particular, T25-MspA interacts with T18-SpsB and MspA-T18 interacts with T25-SpsB. The three sets of plates from left to right represent three biological replicates. pKT25 and pUT18 plasmids were co-transformed as negative control (-) and pKT25 – zip and pUT18C – zip as a positive control (+).


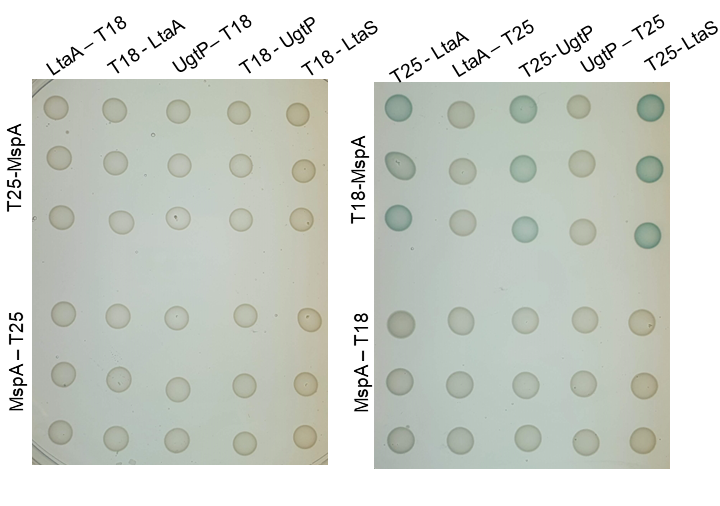


**Figure S8. All bacterial two-hybrid interactions tested between MspA and UgtP, LtaA and LtaS.** MspA (amino acids 25-105) tagged at the C-terminus with T18 interacts with UgtP, LtaA and LtaS when these are tagged at the N-terminus. LtaS was only tagged at the N-terminus as the C-terminus is known to localise extracellularly. Each dot for each combination of interactions tested represents a biological replicate.


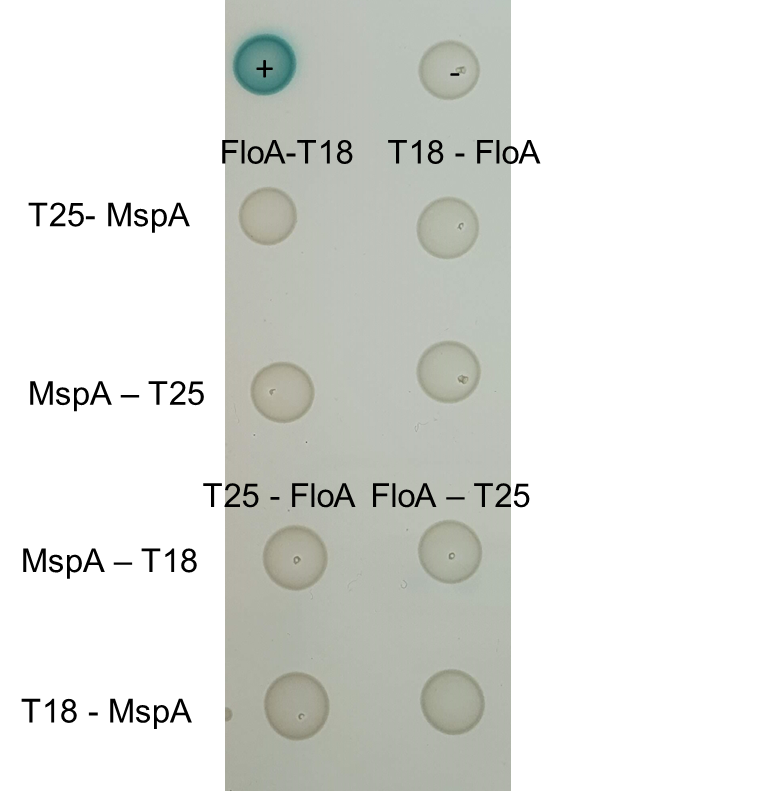


**Figure S9. MspA does not interact with flotillin.** Bacteria two-hybrid assay to test the interaction between MspA and flotillin (FloA). pKT25 and pUT18 plasmids were co-transformed as negative control (-) and pKT25 – zip and pUT18C – zip as a positive control (+). Representative of three biological replicates.


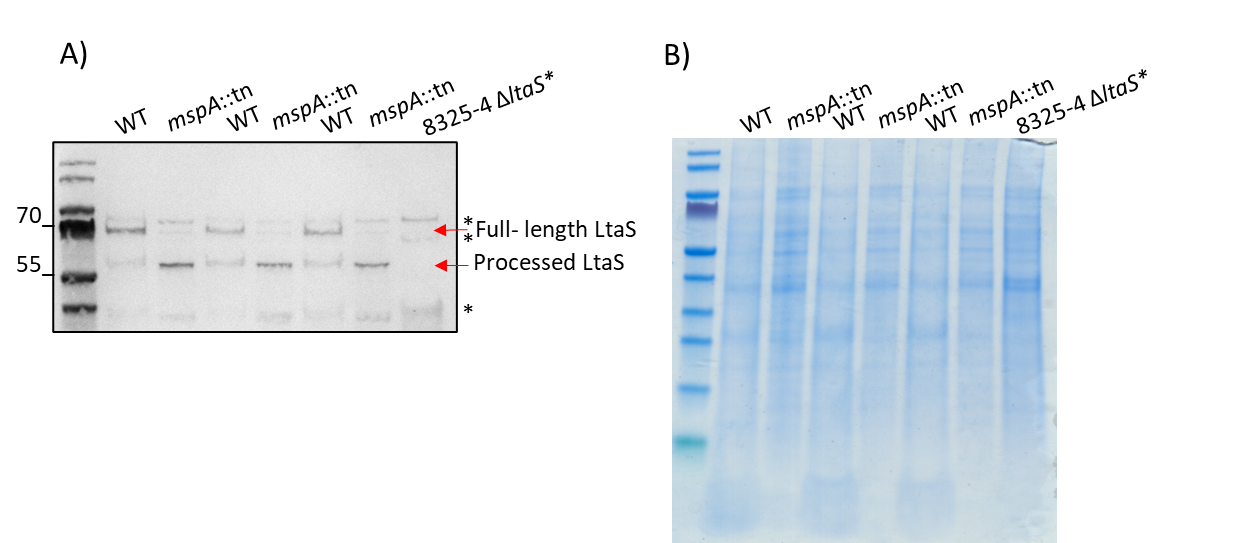


**Figure S10. The MspA-deficient cells displays altered LtaS processing.** A) Western blot with anti-eLtaS antibody on samples from SH1000, SH1000 *mspA*::tn and 8325-4 Δ*ltaS** . Bands deemed as aspecific as they were detected in the *ltaS* knock-out strain are indicated with an asterisk (*). The bands corresponding to full-length LtaS (70 kDa) and the eLtaS processed extracellular domain (55 kDa) are indicated with red arrows. Each pair of samples represents an individual biological replicate. B) Coomassie stain of SDS-PAGE showing total protein loading control.

**Table S1. Proteins significantly enriched with MspA-mCherry in a co-immunoprecipitation.** Proteins in the co-IP elution fractions were identified by LC-MS. The abundance of each protein in the elution fraction of the sample where MspA-mCherry was used as a bait was compared to the abundance of the same protein in the elution fraction obtained using the negative control strain (SH1000 pCN34). Proteins were considered significantly enriched with MspA-mCherry if log(p-value)> 1.3 and log_2_(abundance fold-change) > 2.

| **Accession** | **Description** |
| --- | --- |
| MspA mCherry | MspA mCherry bait |
| Q2FXJ6 | Serine protease HtrA-like OS=Staphylococcus aureus (strain NCTC 8325 / PS 47) OX=93061 GN=SAOUHSC_01838 PE=3 SV=1 |
| Q2FXF4 | Mannosyl-glycoprotein endo-beta-N-acetylglucosamidase-like domain-containing protein OS=Staphylococcus aureus (strain NCTC 8325 / PS 47) OX=93061 GN=SAOUHSC_01895 PE=1 SV=1 |
| Q2FWA8 | Lytic regulatory protein, putative OS=Staphylococcus aureus (strain NCTC 8325 / PS 47) OX=93061 GN=SAOUHSC_02390 PE=4 SV=1 |
| Q2G0R0 | ATP-dependent zinc metalloprotease FtsH OS=Staphylococcus aureus (strain NCTC 8325 / PS 47) OX=93061 GN=ftsH PE=3 SV=1 |
| Q2FZJ9 | Probable quinol oxidase subunit 2 OS=Staphylococcus aureus (strain NCTC 8325 / PS 47) OX=93061 GN=qoxA PE=3 SV=1 |
| Q2FW93 | YbbR-like domain-containing protein OS=Staphylococcus aureus (strain NCTC 8325 / PS 47) OX=93061 GN=SAOUHSC_02406 PE=4 SV=1 |
| Q2G1M2 | Uncharacterized protein OS=Staphylococcus aureus (strain NCTC 8325 / PS 47) OX=93061 GN=SAOUHSC_00085 PE=4 SV=1 |
| Q2FW64 | Alkaline shock response membrane anchor protein AmaP OS=Staphylococcus aureus (strain NCTC 8325 / PS 47) OX=93061 GN=SAOUHSC_02443 PE=4 SV=1 |
| Q2FZT7 | Signal peptidase I OS=Staphylococcus aureus (strain NCTC 8325 / PS 47) OX=93061 GN=SAOUHSC_00903 PE=3 SV=1 |
| Q2FZK3 | Teichoic acid D-alanine hydrolase OS=Staphylococcus aureus (strain NCTC 8325 / PS 47) OX=93061 GN=fmtA PE=2 SV=1 |
| Q2FXI0 | D-alanine aminotransferase OS=Staphylococcus aureus (strain NCTC 8325 / PS 47) OX=93061 GN=SAOUHSC_01867 PE=3 SV=1 |
| Q2FY21 | Penicillin-binding protein 3 OS=Staphylococcus aureus (strain NCTC 8325 / PS 47) OX=93061 GN=SAOUHSC_01652 PE=3 SV=1 |
| Q2G2H3 | ABC transporter domain-containing protein OS=Staphylococcus aureus (strain NCTC 8325 / PS 47) OX=93061 GN=SAOUHSC_02009 PE=4 SV=1 |
| Q2G2I1 | Membrane-associated protein TcaA OS=Staphylococcus aureus (strain NCTC 8325 / PS 47) OX=93061 GN=tcaA PE=2 SV=1 |
| Q2FYL3 | Probable CtpA-like serine protease OS=Staphylococcus aureus (strain NCTC 8325 / PS 47) OX=93061 GN=SAOUHSC_01427 PE=3 SV=1 |
| Q2FY36 | Rhodanese domain-containing protein OS=Staphylococcus aureus (strain NCTC 8325 / PS 47) OX=93061 GN=SAOUHSC_01630 PE=4 SV=1 |
| Q2FZW3 | Protein DltD OS=Staphylococcus aureus (strain NCTC 8325 / PS 47) OX=93061 GN=dltD PE=1 SV=1 |
| Q2FYI0 | Penicillin-binding protein 2 OS=Staphylococcus aureus (strain NCTC 8325 / PS 47) OX=93061 GN=SAOUHSC_01467 PE=4 SV=1 |
| Q2G2S0 | Adenylosuccinate lyase OS=Staphylococcus aureus (strain NCTC 8325 / PS 47) OX=93061 GN=purB PE=3 SV=1 |
| Q2G2W5 | Membrane fusion protein biotin-lipoyl like domain-containing protein OS=Staphylococcus aureus (strain NCTC 8325 / PS 47) OX=93061 GN=SAOUHSC_02630 PE=4 SV=1 |
| Q2FZ64 | non-specific serine/threonine protein kinase OS=Staphylococcus aureus (strain NCTC 8325 / PS 47) OX=93061 GN=SAOUHSC_01187 PE=4 SV=1 |
| Q2FZ94 | Penicillin-binding protein 1 OS=Staphylococcus aureus (strain NCTC 8325 / PS 47) OX=93061 GN=SAOUHSC_01145 PE=1 SV=1 |
| Q2G2X6 | Penicillin-binding protein 4, putative OS=Staphylococcus aureus (strain NCTC 8325 / PS 47) OX=93061 GN=SAOUHSC_00646 PE=1 SV=1 |
| Q2FVV8 | Transcriptional regulator, putative OS=Staphylococcus aureus (strain NCTC 8325 / PS 47) OX=93061 GN=SAOUHSC_02583 PE=3 SV=1 |
| Q2FVN6 | DUF4889 domain-containing protein OS=Staphylococcus aureus (strain NCTC 8325 / PS 47) OX=93061 GN=SAOUHSC_02666 PE=4 SV=1 |
| Q2FVC5 | Uncharacterized lipoprotein SAOUHSC_02788 OS=Staphylococcus aureus (strain NCTC 8325 / PS 47) OX=93061 GN=SAOUHSC_02788 PE=3 SV=1 |
| P52078 | Uncharacterized protein SAOUHSC_00997 OS=Staphylococcus aureus (strain NCTC 8325 / PS 47) OX=93061 GN=SAOUHSC_00997 PE=3 SV=2 |
| Q2G257 | Zinc metallopeptidase OS=Staphylococcus aureus (strain NCTC 8325 / PS 47) OX=93061 GN=SAOUHSC_01477 PE=4 SV=1 |
| Q2FW65 | Small integral membrane protein-protein OS=Staphylococcus aureus (strain NCTC 8325 / PS 47) OX=93061 GN=SAOUHSC_02442 PE=4 SV=1 |
| Q2FWE9 | ATP synthase gamma chain OS=Staphylococcus aureus (strain NCTC 8325 / PS 47) OX=93061 GN=atpG PE=3 SV=1 |
| Q2FYV8 | Thermonuclease OS=Staphylococcus aureus (strain NCTC 8325 / PS 47) OX=93061 GN=SAOUHSC_01316 PE=4 SV=1 |
| Q2FVN4 | DUF3139 domain-containing protein OS=Staphylococcus aureus (strain NCTC 8325 / PS 47) OX=93061 GN=SAOUHSC_02668 PE=4 SV=1 |
| Q2G2S8 | Uncharacterized protein YhaN AAA domain-containing protein OS=Staphylococcus aureus (strain NCTC 8325 / PS 47) OX=93061 GN=SAOUHSC_01974 PE=4 SV=1 |
| Q2FWE7 | ATP synthase subunit delta OS=Staphylococcus aureus (strain NCTC 8325 / PS 47) OX=93061 GN=atpH PE=3 SV=1 |
| Q2FWX6 | Exported protein OS=Staphylococcus aureus (strain NCTC 8325 / PS 47) OX=93061 GN=SAOUHSC_02145 PE=4 SV=1 |
| Q2FZG1 | SCP-like extracellular protein OS=Staphylococcus aureus (strain NCTC 8325 / PS 47) OX=93061 GN=SAOUHSC_01069 PE=4 SV=1 |
| Q2FXT8 | Multifunctional fusion protein OS=Staphylococcus aureus (strain NCTC 8325 / PS 47) OX=93061 GN=secD PE=3 SV=1 |
| Q2FZC7 | succinate dehydrogenase OS=Staphylococcus aureus (strain NCTC 8325 / PS 47) OX=93061 GN=SAOUHSC_01105 PE=3 SV=1 |
| Q2FZ08 | Ribonuclease Y OS=Staphylococcus aureus (strain NCTC 8325 / PS 47) OX=93061 GN=rny PE=1 SV=1 |
| Q2G2S7 | Calcineurin-like phosphoesterase domain-containing protein OS=Staphylococcus aureus (strain NCTC 8325 / PS 47) OX=93061 GN=SAOUHSC_01975 PE=4 SV=1 |
| Q2FWW9 | ABC transporter, ATP-binding protein, putative OS=Staphylococcus aureus (strain NCTC 8325 / PS 47) OX=93061 GN=SAOUHSC_02152 PE=1 SV=1 |
| Q2FXU3 | Probable cell wall amidase LytH OS=Staphylococcus aureus (strain NCTC 8325 / PS 47) OX=93061 GN=lytH PE=1 SV=1 |
| Q2G034 | DUF4887 domain-containing protein OS=Staphylococcus aureus (strain NCTC 8325 / PS 47) OX=93061 GN=SAOUHSC_00793 PE=4 SV=1 |
| Q2FVI6 | Uncharacterized protein OS=Staphylococcus aureus (strain NCTC 8325 / PS 47) OX=93061 GN=SAOUHSC_02724 PE=4 SV=1 |
| Q2G239 | Mannitol-specific phosphotransferase enzyme IIA component OS=Staphylococcus aureus (strain NCTC 8325 / PS 47) OX=93061 GN=SAOUHSC_00708 PE=4 SV=1 |
| Q2FVL4 | Amino acid ABC transporter, ATP-binding protein, putative OS=Staphylococcus aureus (strain NCTC 8325 / PS 47) OX=93061 GN=SAOUHSC_02697 PE=4 SV=1 |
| Q2FXK8 | Septation ring formation regulator EzrA OS=Staphylococcus aureus (strain NCTC 8325 / PS 47) OX=93061 GN=ezrA PE=3 SV=1 |
| Q2FV99 | Sortase A OS=Staphylococcus aureus (strain NCTC 8325 / PS 47) OX=93061 GN=srtA PE=1 SV=1 |
| Q2FWE8 | ATP synthase subunit alpha OS=Staphylococcus aureus (strain NCTC 8325 / PS 47) OX=93061 GN=atpA PE=3 SV=1 |
| Q2FZF9 | Glycerophosphoryl diester phosphodiesterase, putative OS=Staphylococcus aureus (strain NCTC 8325 / PS 47) OX=93061 GN=SAOUHSC_01071 PE=4 SV=1 |
| Q2G071 | Fe/B12 periplasmic-binding domain-containing protein OS=Staphylococcus aureus (strain NCTC 8325 / PS 47) OX=93061 GN=SAOUHSC_00749 PE=4 SV=1 |
| Q2FW75 | ABC transporter periplasmic binding protein, putative OS=Staphylococcus aureus (strain NCTC 8325 / PS 47) OX=93061 GN=SAOUHSC_02430 PE=4 SV=1 |
| Q2FYR2 | Aminoacyltransferase FemA OS=Staphylococcus aureus (strain NCTC 8325 / PS 47) OX=93061 GN=femA PE=1 SV=1 |
| Q2FXQ1 | 50S ribosomal protein L20 OS=Staphylococcus aureus (strain NCTC 8325 / PS 47) OX=93061 GN=rplT PE=1 SV=1 |
| Q2FW00 | Sugar transporter, putative OS=Staphylococcus aureus (strain NCTC 8325 / PS 47) OX=93061 GN=SAOUHSC_02520 PE=3 SV=1 |
| Q2G1K8 | Capsular polysaccharide biosynthesis protein, putative OS=Staphylococcus aureus (strain NCTC 8325 / PS 47) OX=93061 GN=SAOUHSC_00114 PE=3 SV=1 |
| Q2G2F3 | Signal transduction protein TRAP OS=Staphylococcus aureus (strain NCTC 8325 / PS 47) OX=93061 GN=traP PE=1 SV=1 |
| Q2FYZ4 | Aerobic glycerol-3-phosphate dehydrogenase OS=Staphylococcus aureus (strain NCTC 8325 / PS 47) OX=93061 GN=glpD PE=3 SV=1 |
| Q2G2S6 | Foldase protein PrsA OS=Staphylococcus aureus (strain NCTC 8325 / PS 47) OX=93061 GN=prsA PE=3 SV=1 |
| Q2FV16 | Probable malate:quinone oxidoreductase OS=Staphylococcus aureus (strain NCTC 8325 / PS 47) OX=93061 GN=mqo PE=3 SV=1 |
| Q2FZC8 | succinate dehydrogenase OS=Staphylococcus aureus (strain NCTC 8325 / PS 47) OX=93061 GN=SAOUHSC_01104 PE=3 SV=1 |
| Q2G1V4 | ABC transporter, ATP-binding protein, putative OS=Staphylococcus aureus (strain NCTC 8325 / PS 47) OX=93061 GN=SAOUHSC_00333 PE=4 SV=1 |
| Q2G0D6 | DUF47 domain-containing protein OS=Staphylococcus aureus (strain NCTC 8325 / PS 47) OX=93061 GN=SAOUHSC_00669 PE=3 SV=1 |
| Q2G2L1 | Teichoic acids export ATP-binding protein TagH OS=Staphylococcus aureus (strain NCTC 8325 / PS 47) OX=93061 GN=tagH PE=3 SV=1 |
| Q2FYR1 | Aminoacyltransferase FemB OS=Staphylococcus aureus (strain NCTC 8325 / PS 47) OX=93061 GN=femB PE=1 SV=1 |
| Q2FWE5 | Serine hydroxymethyltransferase OS=Staphylococcus aureus (strain NCTC 8325 / PS 47) OX=93061 GN=glyA PE=3 SV=1 |
| Q2FYF1 | Elastin-binding protein EbpS OS=Staphylococcus aureus (strain NCTC 8325 / PS 47) OX=93061 GN=ebpS PE=1 SV=1 |
| Q2G2G0 | DM13 domain-containing protein OS=Staphylococcus aureus (strain NCTC 8325 / PS 47) OX=93061 GN=SAOUHSC_00717 PE=4 SV=1 |
| Q2FW06 | 50S ribosomal protein L3 OS=Staphylococcus aureus (strain NCTC 8325 / PS 47) OX=93061 GN=rplC PE=1 SV=1 |
| Q2G2D8 | ABC transporter, substrate-binding protein, putative OS=Staphylococcus aureus (strain NCTC 8325 / PS 47) OX=93061 GN=SAOUHSC_00634 PE=3 SV=1 |
| Q2FZQ3 | Enoyl-[acyl-carrier-protein] reductase [NADPH] FabI OS=Staphylococcus aureus (strain NCTC 8325 / PS 47) OX=93061 GN=fabI PE=1 SV=1 |
| Q2FZH7 | DUF697 domain-containing protein OS=Staphylococcus aureus (strain NCTC 8325 / PS 47) OX=93061 GN=SAOUHSC_01027 PE=4 SV=1 |
| Q2FXM9 | Pyruvate kinase OS=Staphylococcus aureus (strain NCTC 8325 / PS 47) OX=93061 GN=pyk PE=3 SV=1 |
| P0A0F8 | 50S ribosomal protein L15 OS=Staphylococcus aureus (strain NCTC 8325 / PS 47) OX=93061 GN=rplO PE=1 SV=1 |
| Q2FWD1 | CTP synthase OS=Staphylococcus aureus (strain NCTC 8325 / PS 47) OX=93061 GN=pyrG PE=3 SV=1 |
| Q2FW16 | 50S ribosomal protein L14 OS=Staphylococcus aureus (strain NCTC 8325 / PS 47) OX=93061 GN=rplN PE=1 SV=1 |
| Q2FV86 | Pyruvate oxidase, putative OS=Staphylococcus aureus (strain NCTC 8325 / PS 47) OX=93061 GN=SAOUHSC_02849 PE=3 SV=1 |
| O06446 | Protein translocase subunit SecA 1 OS=Staphylococcus aureus (strain NCTC 8325 / PS 47) OX=93061 GN=secA1 PE=1 SV=2 |
| Q2FXM8 | ATP-dependent 6-phosphofructokinase OS=Staphylococcus aureus (strain NCTC 8325 / PS 47) OX=93061 GN=pfkA PE=1 SV=2 |
| Q2FUX7 | Arginine deiminase OS=Staphylococcus aureus (strain NCTC 8325 / PS 47) OX=93061 GN=arcA PE=3 SV=1 |
| Q2FZ89 | Cell division protein FtsZ OS=Staphylococcus aureus (strain NCTC 8325 / PS 47) OX=93061 GN=ftsZ PE=1 SV=1 |
| Q2FWA0 | Glutamine--fructose-6-phosphate aminotransferase [isomerizing] OS=Staphylococcus aureus (strain NCTC 8325 / PS 47) OX=93061 GN=glmS PE=3 SV=1 |
| Q2FYG1 | Glycerol-3-phosphate dehydrogenase [NAD(P)+] OS=Staphylococcus aureus (strain NCTC 8325 / PS 47) OX=93061 GN=gpsA PE=3 SV=1 |
| Q2G0P5 | ATP-dependent Clp protease ATP-binding subunit ClpC OS=Staphylococcus aureus (strain NCTC 8325 / PS 47) OX=93061 GN=clpC PE=1 SV=1 |
| Q2FZ28 | ATP-dependent protease ATPase subunit HslU OS=Staphylococcus aureus (strain NCTC 8325 / PS 47) OX=93061 GN=hslU PE=3 SV=1 |
| Q2FVF4 | Glutamate synthase alpha subunit, putative OS=Staphylococcus aureus (strain NCTC 8325 / PS 47) OX=93061 GN=SAOUHSC_02760 PE=3 SV=1 |
| Q2FZ27 | Global transcriptional regulator CodY OS=Staphylococcus aureus (strain NCTC 8325 / PS 47) OX=93061 GN=codY PE=1 SV=1 |
| Q2FZV7 | Type II NADH:quinone oxidoreductase OS=Staphylococcus aureus (strain NCTC 8325 / PS 47) OX=93061 GN=SAOUHSC_00878 PE=1 SV=1 |
| Q2G245 | Smooth muscle caldesmon OS=Staphylococcus aureus (strain NCTC 8325 / PS 47) OX=93061 GN=SAOUHSC_01854 PE=4 SV=1 |
| Q2G2A3 | Dihydrolipoyl dehydrogenase OS=Staphylococcus aureus (strain NCTC 8325 / PS 47) OX=93061 GN=SAOUHSC_01043 PE=3 SV=1 |
| Q2FW31 | 30S ribosomal protein S11 OS=Staphylococcus aureus (strain NCTC 8325 / PS 47) OX=93061 GN=rpsK PE=1 SV=1 |
| Q2FW11 | 50S ribosomal protein L22 OS=Staphylococcus aureus (strain NCTC 8325 / PS 47) OX=93061 GN=rplV PE=1 SV=1 |
| Q2FZ55 | Phosphate acyltransferase OS=Staphylococcus aureus (strain NCTC 8325 / PS 47) OX=93061 GN=plsX PE=3 SV=1 |
| Q2FW23 | 30S ribosomal protein S5 OS=Staphylococcus aureus (strain NCTC 8325 / PS 47) OX=93061 GN=rpsE PE=1 SV=1 |
| Q2G115 | Ribosome-binding ATPase YchF OS=Staphylococcus aureus (strain NCTC 8325 / PS 47) OX=93061 GN=ychF PE=3 SV=1 |
| Q2FXI5 | Glutamyl aminopeptidase OS=Staphylococcus aureus (strain NCTC 8325 / PS 47) OX=93061 GN=SAOUHSC_01861 PE=3 SV=1 |
| Q2FW18 | 50S ribosomal protein L5 OS=Staphylococcus aureus (strain NCTC 8325 / PS 47) OX=93061 GN=rplE PE=1 SV=1 |
| Q2G2M3 | Putative TrmH family tRNA/rRNA methyltransferase OS=Staphylococcus aureus (strain NCTC 8325 / PS 47) OX=93061 GN=SAOUHSC_00513 PE=4 SV=1 |
| Q2G0P0 | 50S ribosomal protein L1 OS=Staphylococcus aureus (strain NCTC 8325 / PS 47) OX=93061 GN=rplA PE=3 SV=1 |
| Q2FZ25 | 30S ribosomal protein S2 OS=Staphylococcus aureus (strain NCTC 8325 / PS 47) OX=93061 GN=rpsB PE=1 SV=2 |
| Q2FWF5 | 3-hydroxyacyl-[acyl-carrier-protein] dehydratase FabZ OS=Staphylococcus aureus (strain NCTC 8325 / PS 47) OX=93061 GN=fabZ PE=3 SV=1 |
| Q2FWM4 | Accessory gene regulator protein A OS=Staphylococcus aureus (strain NCTC 8325 / PS 47) OX=93061 GN=SAOUHSC_02265 PE=4 SV=1 |
| Q2FZK7 | Bifunctional autolysin OS=Staphylococcus aureus (strain NCTC 8325 / PS 47) OX=93061 GN=atl PE=1 SV=1 |
